# Automated eyeball volume measurement based on CT images using neural network-based segmentation and simple estimation

**DOI:** 10.1038/s41598-024-64913-9

**Published:** 2024-07-02

**Authors:** Sujeong Han, Jeong Kyu Lee, Daewon Lee, Jaesung Lee

**Affiliations:** 1 Department of Artificial Intelligence, Chung-Ang University, Seoul, 06974 Republic of Korea; 2 Department of Ophthalmology, Chung-Ang University College of Medicine, Chung-Ang University Hospital, Seoul, 06973 Republic of Korea; 3 Department of Art and Technology, Chung-Ang University, Anseong, 17546 Republic of Korea

**Keywords:** Biomedical engineering, Eye manifestations

## Abstract

With the increase in the dependency on digital devices, the incidence of myopia, a precursor of various ocular diseases, has risen significantly. Because myopia and eyeball volume are related, myopia progression can be monitored through eyeball volume estimation. However, existing methods are limited because the eyeball shape is disregarded during estimation. We propose an automated eyeball volume estimation method from computed tomography images that incorporates prior knowledge of the actual eyeball shape. This study involves data preprocessing, image segmentation, and volume estimation steps, which include the truncated cone formula and integral equation. We obtained eyeball image masks using U-Net, HFCN, DeepLab v3 +, SegNet, and HardNet-MSEG. Data from 200 subjects were used for volume estimation, and manually extracted eyeball volumes were used for validation. U-Net outperformed among the segmentation models, and the proposed volume estimation method outperformed comparative methods on all evaluation metrics, with a correlation coefficient of 0.819, mean absolute error of 0.640, and mean squared error of 0.554. The proposed method surpasses existing methods, provides an accurate eyeball volume estimation for monitoring the progression of myopia, and could potentially aid in the diagnosis of ocular diseases. It could be extended to volume estimation of other ocular structures.

## Introduction

The rapid advancement of science and technology has led to the excessive use of digital devices, such as smartphones and computers. This phenomenon has accelerated the aging of the eyes and resulted in a significant increase in the incidence of ocular disorders^1–3^. Myopia is the most common ocular disorder and is often characterized by excessive elongation of the eyeball axial length^4–6^. Because axial length and eyeball volume estimation are correlated, calculating the axial length using eyeball volume estimation can provide valuable information for tracking the progression of myopia. Various ocular complications are associated with myopia, including retinal detachment, glaucoma, cataracts, and macular degeneration, and are contingent upon the severity of progression^7–9^. The estimation of eyeball volume can provide useful information to clinicians in terms of ocular anomaly identification and ocular disease diagnosis. Moreover, it can provide valuable information for monitoring the outbreak of eye-related complications and assist in planning treatment or surgery^10–13^. Therefore, eyeball volume estimation is essential for ocular health diagnosis and the prevention of ocular complications.

Traditionally, eyeball volume has been estimated using methods relying on intraocular pressure measurements or specialized software for medical imaging^12,14,15^. However, these methods rely on specialized equipment or invasive medical procedures and thus are not only time-consuming and costly but also pose a risk of infection. With the development of medical imaging techniques, measuring the eyeball volume using various medical imaging techniques such as radiography, ultrasound, computed tomography (CT), magnetic resonance imaging, and photography have been researched for a long time. However, these methods face limitations such as time-consuming manual procedures and accuracy dependent on the expertise of clinicians and have not been widely applied clinically^16–19^. Recent advancements in artificial intelligence have prompted attempts to estimate eyeball volume based on medical imaging using deep learning^20^. However, limitations to accurately estimating eyeball volume remain because the shape of the eyeball is ignored during the estimation process. In this study, we propose an eyeball volume estimation template that incorporates prior information to reflect the actual shape of the eyeball.

## Results

The performance of each image segmentation model is shown in Table 1. As a result of the eyeball segmentation experiment, all segmentation models showed reasonable performance. This can be interpreted because the position of the eyeball on the CT image is consistent, and in the data processing, we selectively used only slices where the boundary of eyeball was clearly visible. Finally, we selected U-Net as the segmentation model owing to its highest performance with mean Dice score of 0.952 and mean IoU of 0.912. Most of the mask images from the segmentation model were circular. (See Fig. S1).

**Table 1:** Performance of segmentation models.

Model	IoU	Dice
U-Net	0.912 ± 0.033	0.952 ± 0.020
HFCN	0.909 ± 0.030	0.940 ± 0.019
DeepLab V3 +	0.911 ± 0.033	0.951 ± 0.020
SegNet	0.906 ± 0.035	0.947 ± 0.021
HardNet-MSEG	0.906 ± 0.031	0.948 ± 0.019

The performance of each eyeball volume estimation method is presented in Table 2. Three metrics were determined to evaluate the similarity between each method and the manually calculated volume. The proposed method performed better than the comparative models in all metrics, with a Corr of 0.819, MAE of 0.640, and MSE of 0.554 for the eyeball volume estimation. These experimental results demonstrate the effectiveness of fully considering the eyeball shape in terms of estimation performance.

**Table 2:** Performance of volume estimation methods.

	Corr	MAE	MSE
Proposed method	0.819	0.640	0.554
Fangzhou et al.(2019)	0.769	0.684	0.710
Pixel Count	0.763	0.693	0.729

## Materials and methods

This study is a retrospective comparative effectiveness research study. The protocol was approved by the institutional review board of the Chung-Ang University Hospital (IRB No. 2311-017-19498) and complies with the tenets of the Declaration of Helsinki. The requirement for informed consent was waived by the institutional review board because of the retrospective nature of the study.

Existing methods for estimating eyeball volume have relied on specialized equipment or invasive medical procedures. However, these approaches are constrained not only in terms of time and cost but also pose limitations in patient comfort and carry the risk of infection. To address this issue, there have been efforts to estimate volume using a deep learning-based approach utilizing CT image segmentation. While this overcomes the limitations of traditional methods, it is significantly influenced by the performance of the segmentation model. Although these methods overcome the limitations of traditional methods, it is significantly influenced by the performance of the segmentation model. These methods rely heavily on pixel-based estimations of the eyeball volume, making it challenging to accurately reflect the actual shape of the eyeball. This leads to an increase in volume estimation errors. However, incorporating a template that reflects the actual shape of eyeball, it can effectively reduce the margin of error in volume estimation.

### Image preprocessing

**Figure 1:**
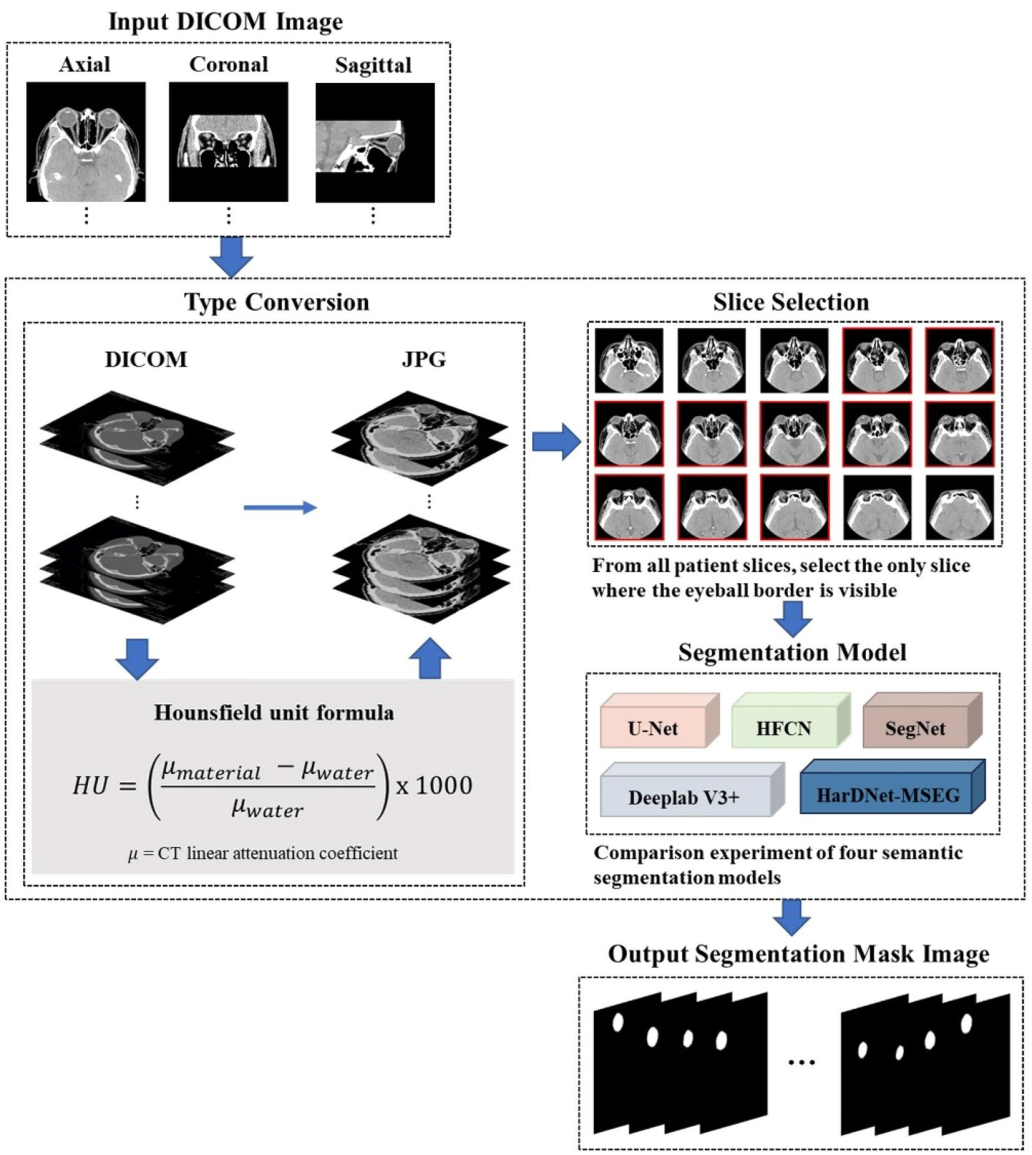
Procedure of image segmentation framework with data preprocessing.

Figure 1 shows the image segmentation framework with preprocessing. In this study, we constructed a CT image dataset and extracted metadata from the Digital Imaging and Communications in Medicine (DICOM) files. DICOM is a data storage format used in the medical field to store medical images such as CT and MRI along with image information and patient data. CT images were quantified in Hounsfield units (HU), which indicate the degree of X-ray absorption in the body. Hence, by adjusting the HU values using two parameters, one can emphasize specific regions of interest. Among the two parameters for HU value adjustment, window center focuses on the HU value to be targeted, while window width indicates the range of HU values to be observed around the window center. Through empirical studies, we determined the optimal values for HU adjustment to effectively identify the eyeball in CT slices. Following the above procedure, we manually selected CT slices with distinctly visible eyeball to ensure good image segmentation performance. This step is essential to use only the images where the eyeball is clearly visible for training and prediction of the segmentation model.

### Image segmentation

We conducted a comparative experiment by implementing five segmentation models: U-Net^21^, HFCN^22^, DeepLab v3+^23^, SegNet^24^, and HardNet-MSEG^25^. For detailed information on the segmentation models used in the experiment, please refer to the supplementary information. Throughout the entire training process, we ensured reasonable performance through iterative training of the segmentation models. Among several segmentation models, we selected the model demonstrating the best performance. Ultimately, we leveraged the best-performing image segmentation model to obtain mask images for CT images of all patients, thereby enabling the construction of the final mask image dataset.

### Volume estimation

Image segmentation is essential for identifying the boundaries of complex biological structures such as the eyeball. The precise boundaries of these structures obtained through segmentation are important for increasing the accuracy of volume estimation. Please refer to the supplementary information for studies related to volume estimation.

**Figure 2:**
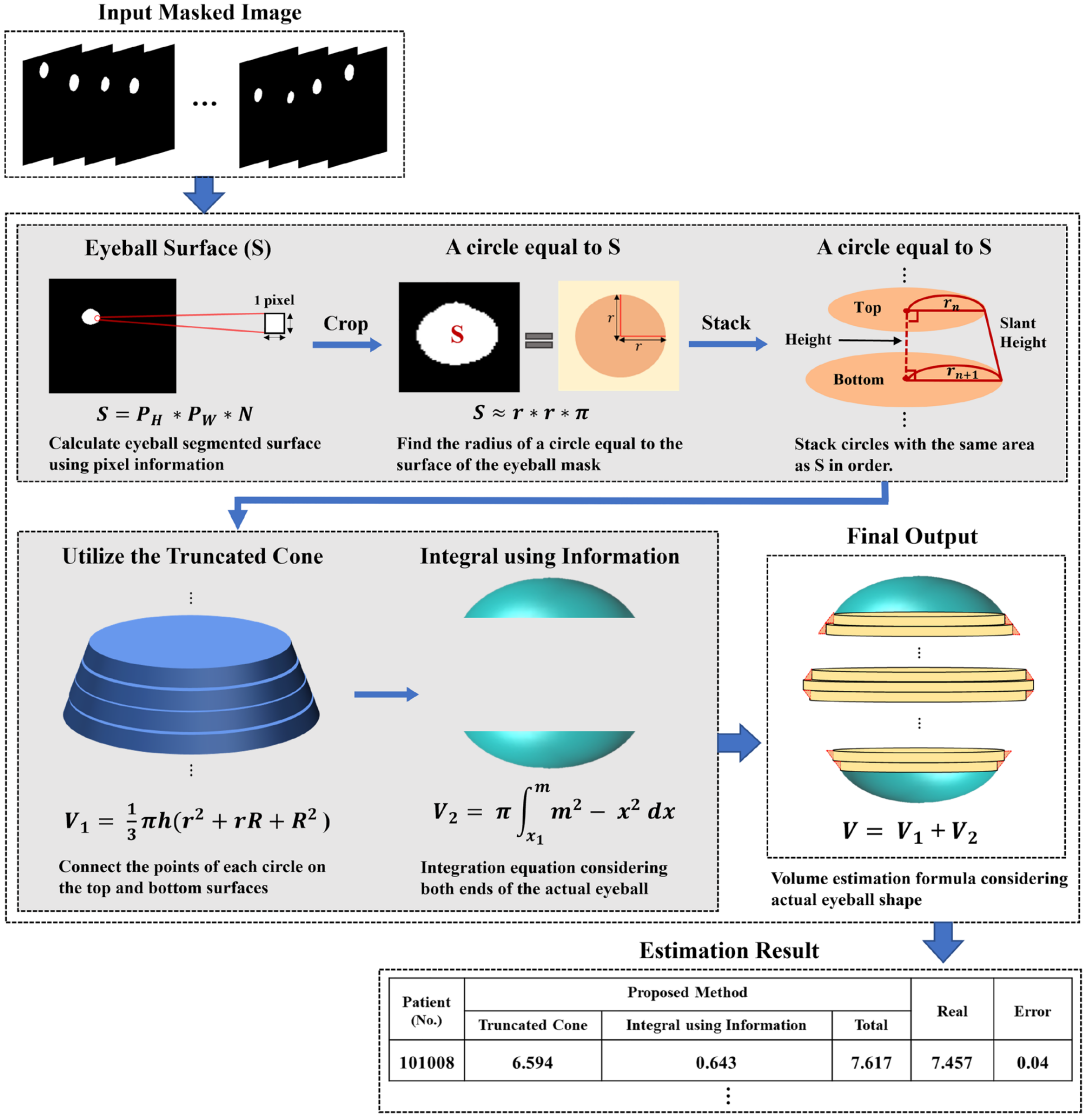
Procedure of the volume estimation framework.

Figure 2 shows the volume estimation framework. We derived the eyeball area in each slice by implementing binarization and mean, considering the established pixel resolution and dimensions (See Fig. S2a). The entire process is formulated as follows: where *I* is the input eyeball region of interest (RoI), *f*_*B*_ is the binarization function, *p*_*w*_ is the physical width of a pixel, *p*_*h*_ is the physical height of a pixel, and *S* is the physical area of the RoI. As the eyeball occupied only a small part of the CT image, we cropped the RoI corresponding to the eyeball from the mask image. By applying a known pixel resolution and image dimensions, we accurately estimated the eyeball area. Eq. (1) represents the function *f*_*B*_ applied to image *I*, resulting in *B* being the binary mask of the RoI.

1 $$\text{B}=f\_B(\text{I})$$

Next, Eq. (2) represents the conversion of pixel area *A* to physical area *S*, using pixel height *p*_*h*_ and pixel width *p*_*w*_ to manually calculate the eyeball area.

2 $$S=A \cdot {p}_{k} \cdot {p}_{w}$$

where *A* = *mean*(*B*) is the average value of *B*, which represents the eyeball area in pixels.

We then calculated the radius of the circle that approximates the manually calculated inferred area of each eyeball (See Fig. S2b). Next, we approximated the eyeball area, S, for satisfying the area formula of the circle as $S \approx \pi *{r}^{2}$ with an approximated radius $r \approx \sqrt{\frac{\pi }{S}}$ from approximated area S. We argue that this is a reasonable approximation for central cross-sections perpendicular to the eyeball, which are typically nearly circular in shape.

Next, we stacked the mask images of each patient in the z-axis direction to estimate the eyeball volume. To accurately estimate the eyeball volume, we utilized both the eyeball area and the spacing between adjacent image slices. Existing pixel-based methods simply use the truncated cylinder formula to stack mask images (See Fig. S2c). The volume estimation process using this formula is as follows.

3 $${V}_{i}=\pi *{r}_{i}^{2}*{h}_{i}$$

where

4 $$\widehat{V} = \sum_{i=1}^{N-1}{V}_{i}$$

As shown in Eq. (3), *V*_*i*_ represents the volume of *i*th slice using the truncated cylinder formula. *r*_*i*_ is the radii estimated from the *i*th slice areas, and *h*_*i*_ is the gap between the *i*th and *i* + 1th consecutive slices. As shown in Eq. (4), the final volume *V*^ˆ^ is calculated by accumulating the estimated volumes for all slices *N*.

However, these pixel-based methods do not reflect the curvature of an actual eyeball. It is difficult to accurately estimate eyeball volume because there is no one-to-one correspondence between the points when the top and bottom sides of the slice are connected by a straight line. To overcome these limitations, we propose an eyeball template-based approach to volume estimation that considers the curvature of an actual eyeball using a truncated cone formula (See Fig. S2d). The volume estimation process using this formula is as follows.

5 $${V}_{i}=\frac{1}{3}\pi *{h}_{i}\left({r}_{i}^{2}+{r}_{i}*{r}_{i+1} + {r}_{i+1}^{2}\right)$$

where

6 $$\widehat{V} = \sum_{i=1}^{N-1}{V}_{i}.$$

In Eq. (5), the volume Vi of the ith slice is computed by the formula for a truncated cone, considering *r*_*i*_ and *r*_*i*+*1*_ to be the radii calculated from the adjacent slices and hi the interslice interval. Next, the overall volume was determined using Eq. (6) by accumulating the volumes from all slices. Due to the limitations of the segmentation model, the volume at the end of the eyeball was not considered. We uses an integral equation reflecting prior knowledge of the eyeball shape to calculate the volume of both ends of the eyeball. (See Fig. S2e) The volume estimation method for both ends of the eyeball is as follows.

7 $${V}_{k}=\pi {\int }_{x1}^{m}{m}^{2}-{x}^{2}dx$$

We derived Eq. (7) from the formula used to calculate the volume of a sphere, which involves the stacking of small cylinders along both ends of the sphere. We obtained the smallest radius x1 and the largest radius m among all slices from both ends and the central part of the eyeball. To determine the volume Vk at both ends of the eyeball, we integrated them from the smallest to the largest radius of the eyeball. The volumes of the two ends of the eyeball were estimated using integral equations, based on the assumption that the eyeball is spherical.

There are two main reasons the eyeball volume estimation performance can be improved using our method. First, we effectively reflected the eyeball shape using a truncated cone formula with the prior knowledge that the eyeball is spherical. Because the estimated eyeball volume is larger when the truncated cone method is used than when the truncated cylinder method is used, the errors can be reduced. This is because the estimated volume is always smaller than the manually calculated volume because the existing process uses only slices in which the eyeball is clearly visible on the CT image. Second, volume estimation errors occur because the ends of the eyeball are not considered. For example, there are 15 CT images of the eyeballs of the patient; however, owing to the limitations of segmentation, only 10 images were used for volume estimation (See Fig. S3). To overcome this limitation, the volumes at both ends of the eyeball were estimated using integral equations considering the eyeball shape. Thus, we can reduce the volume estimation errors. We compared simplified volume estimation of the pixel-based and proposed methods, respectively (See Fig. S4a and b).

## Experiment

### Dataset

We used a CT image dataset comprising orbital CT scans obtained from patients who visited a research cooperative at a university hospital. CT slices in which the eyeball was clearly discernible were selected for each patient, and a 2D CT image dataset of 200 patients was constructed. The dataset comprised 15,818 images with a size of 512 × 512 pixels. The 2D CT image dataset was used to evaluate the performance of the proposed method.

The dataset involves the demographic statistics of the patients whose CT images were collected (see Table S1). The average age of the patients was 35.06 years (range: 10–67 years), with an average age of 34.28 (± 12.54) years for the 65 male subjects and an average age of 35.44 (± 11.35) years for the 135 female subjects.

### Experimental detail

To obtain the eyeball mask images, we selected various comparative models: U-Net, HFCN, DeepLab v3+, SegNet, and HardNet-MSEG. The hyperparameters were set as follows: batch size of 64, 50 epochs, AdamW optimization algorithm, and learning rate of 1e-4. In the volume estimation step, data from the 200 subjects in the CT image dataset were used. For validation, eyeball volumes were manually calculated from CT images by experienced ophthalmologists using TeraRecon software^26^. The performance of the image segmentation models was evaluated using commonly employed metrics in image segmentation: the intersection over union and the Dice coefficient. Various eyeball volume estimation methods, including the proposed method, were evaluated using three evaluation metrics: correlation coefficient (Corr), mean absolute error (MAE), and mean squared error (MSE).

## Discussion

**Figure 3:**
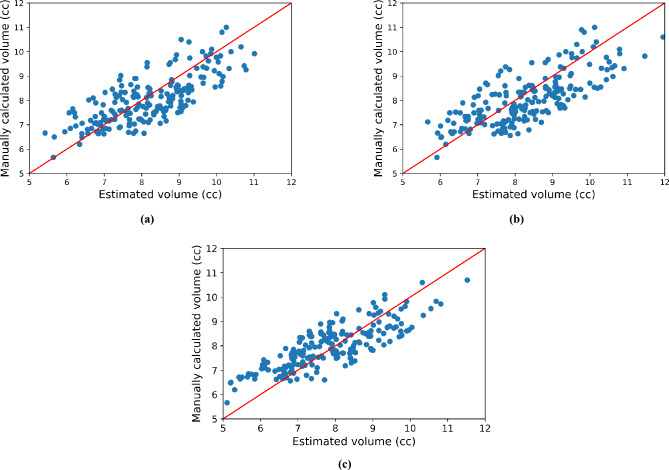
Scatter plot of volume estimation methods. (**a**) Pixel-based method, (**b**) Conventional method, (**c**) Proposed method.

Figure 3a–c show scatter plots of the manually calculated eyeball volume and the eyeball volumes estimated by all comparative methods, respectively. The x-axis of the scatter plot is the eyeball volume predicted by the each method, and the y-axis is the manually calculated eyeball volume. In particular, we can see the linear shape of the scatter plot about the proposed method. It indicates that the proposed method performed well in estimating the eyeball volume.

**Figure 4:**
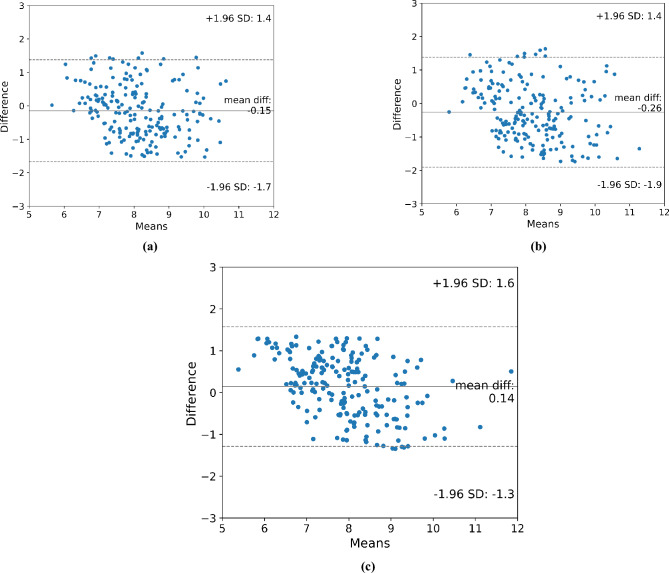
Bland–Altman plot of volume estimation methods. (**a**) Pixel-based method, (**b**) Conventional method, (**c**) Proposed method.

Figure 4a–c show the Bland–Altman plots of the manually calculated eyeball volume and the eyeball volume estimated by all comparative methods, respectively. The Bland–Altman plot shows the extent to which the estimated value differed from the measured value. The degree of discrepancy is indicated by three horizontal lines parallel to the X-axis. The center line represents the mean difference, the top line represents the upper 95% limits of agreement, calculated as the sum of the mean and 1.96*standard deviation, and the bottom line represents the lower 95% limits of agreement, calculated as the sum of the mean and −1.96*standard deviation. The plot of the proposed method does not deviate from the upper and lower limits unlike the other methods, which can be interpreted as the smallest volume estimation error of the proposed method. We observe that the proposed method has a mean difference closer to zero compared to the other methods, indicating a smaller volumetric error. The range of limits of agreement for the proposed method is slightly narrower, suggesting a smaller volumetric error. Additionally, the scatter of points within the limits of agreement for the proposed method (Fig. 4c) appears more concentrated around the mean difference line compared to the other methods. This indicates fewer significant discrepancies in volume estimation. Although the difference is marginal, based on the three perspectives mentioned earlier, we interpreted that our proposed method exhibits the least volumetric measurement error.

**Figure 5:**
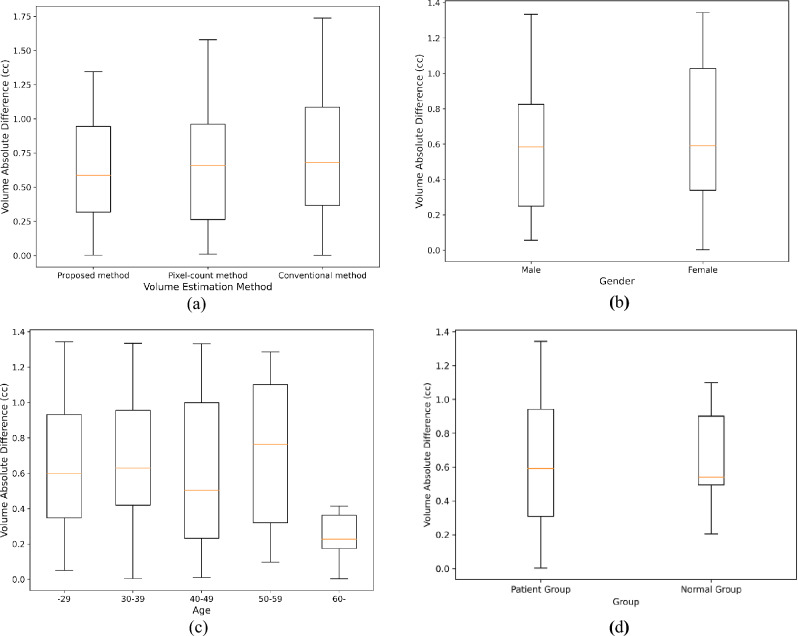
Box plot of volume estimation methods. (**a**) All methods, (**b**) Proposed method by gender, (**c**) Proposed method by age, (**d**) Proposed method by pathological condition.

Figure 5a shows a box plot of the volume errors between the manually calculated eyeball volume and the estimated eyeball volume for all the comparative methods. The box plot is a figure that allows you to easily understand the distribution shape, symmetry, extreme values, etc. of data using quartile values. It is widely used in statistics because it provides an overall view of the data. The median also determines the position of the line in the middle of the box. The box plot of the proposed method can be interpreted as having the best volume estimation because it has the smallest median value and box size.

Figure 5b and c show box plots of the volume errors according to gender and age between the manually calculated eyeball volume and the eyeball volume estimated using the proposed method, respectively. In the case of gender, the margin of error was larger for women than for men. In the case of age, the patterns of the box plots were very different. The smallest error was observed in the eyeball volume estimation of patients aged 60 years and older and the largest error in that of patients aged 50–59 years. This phenomenon is likely related to eye disease and eyeball shape. In fact, Ocular diseases can cause anatomical changes in the eyeball^27^. Abnormal eyeball shapes are likely to increase segmentation errors. According to research on thyroid eye disease (TED), the highest number of patients is in their 50s, rapidly decreasing after the age of 60^28^. Therefore, we consider that people in their 50s have an increased probability of various anatomical changes, which may have resulted in greater eyeball volume estimation errors. On the other hand, it seems that the incidence of ocular diseases decreases after the age of 60, and thus, the estimation error also decreases.

Finally, Fig. 5d shows a box plot of the volume errors according to pathological conditions between the manually calculated eyeball volume and the eyeball volume estimated using the proposed method. This suggests that the proposed eyeball volume measurement method works consistently regardless of group.

**Figure 6:**
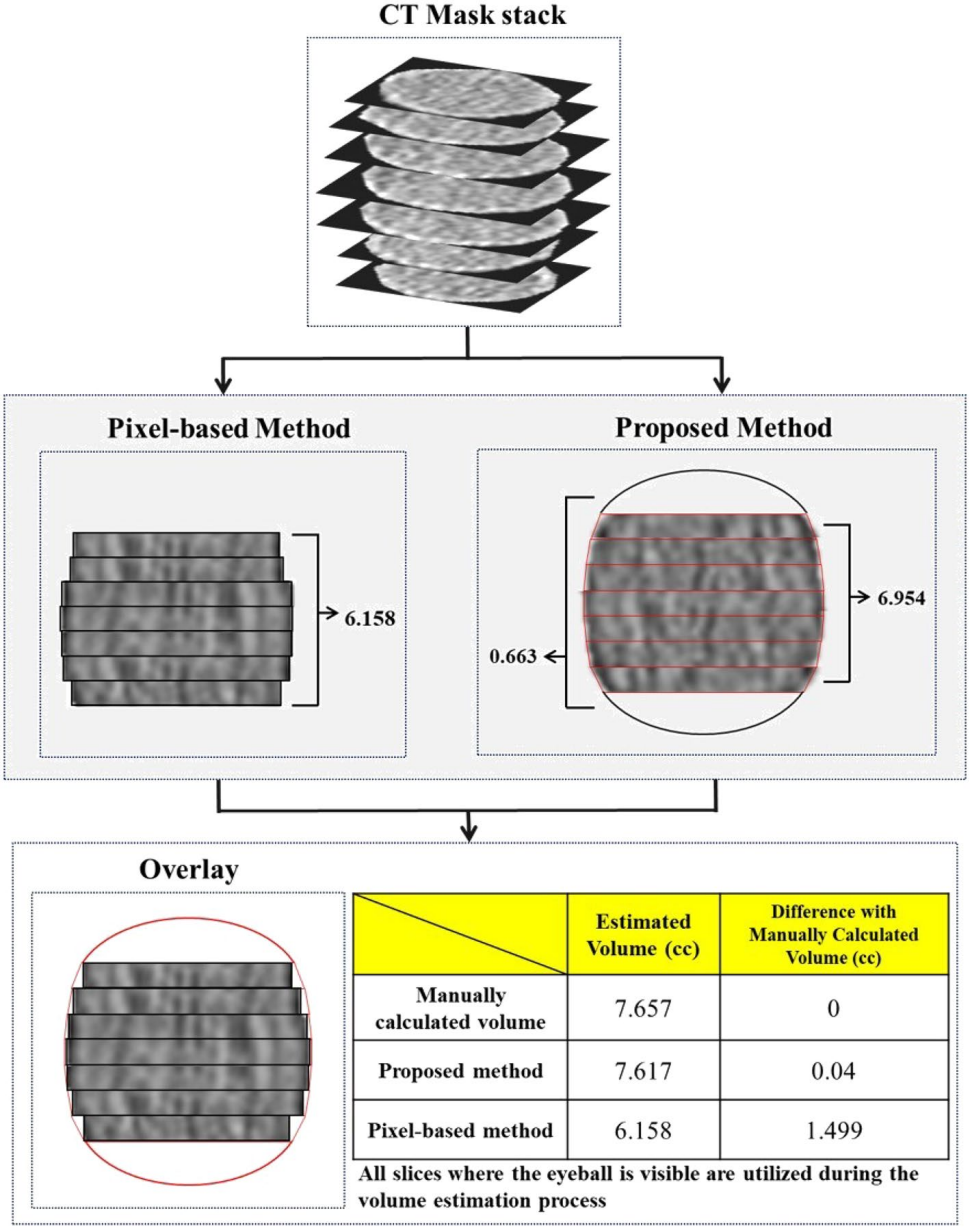
Example of comparison between existing method and proposed methods for estimating volume using CT mask.

Figure 6 shows an example of a volume estimate for comparing the pixel-based and proposed methods. The volume estimated for the pixel-based method is 6.158cc. In addition, the volume estimated for the middle part through the truncated cone formula of the proposed method is 6.954cc, and the volume estimated for both ends through the integral equation is 0.663cc, for a total of 7.617cc. The manually calculated volume of the patient is 7.657cc, and the error with the pixel-based method is 1.499cc and the error with the proposed method is 0.04cc. In the overlay, the red area represents the part where the volume that the pixel-based method could not estimate was supplemented with the proposed method. This indicates that the proposed method overcomes the limitations of the pixel-based method and enables more accurate volume estimation.

In conclusion, eyeball volume estimation is essential in ophthalmology because of its clinical significance. Despite its importance, this field has received relatively low attention, as it does not directly impact human health. Existing methods are unable to provide one-to-one correspondence for all points between consecutive slices; therefore, their volume estimation performance is limited owing to information loss between slices. To overcome this problem, we propose a novel method for eyeball volume estimation that uses a template that incorporates information regarding the actual shape of the eyeball. We used truncated cone and integral formulas to minimize information loss by reflecting the actual shape and curvature of the eyeball. Ultimately, the proposed method exhibited superior performance in terms of eyeball volume estimation across all metrics compared with existing methods.

However, there are still several limitations to the eyeball volume estimation method. First, most of the studies included ours utilized only the slices where the eyeball was clearly visible. We consider that performance errors arising from this exclusion of slices are inevitable. If image segmentation performance improves, it may encompass slices previously excluded from volume estimation. Next, since the volume estimation process of the proposed method involves stacking mask images to estimate the volume, there is a possibility that small errors are accumulated during the stacking process, resulting in performance degradation. Therefore, developing an end-to-end model for direct volume prediction could enable more precise volume estimation. This approach holds the potential for achieving a more accurate estimation by omitting the stacking process, thus mitigating the risk of cumulative errors. We will extend the proposed method to estimate the volumes of other ocular structures in the future and this will potentially aid clinicians in diagnosing thyroid-related ophthalmic conditions.

### Supplementary Information

Supplementary Information.

## Data Availability

Datasets used and analyzed during the current study are available from the corresponding author upon reasonable request.
