# Supplementary material for: Automated eyeball volume measurement based on CT images using neural network-based segmentation and simple estimation
Source: Sci Rep. 2024 Jul 2;14:15094. doi: 10.1038/s41598-024-64913-9 (PMC11219917; doi:10.1038/s41598-024-64913-9)
Supplement: Supplementary file 1 — Supplementary Information. [file 41598_2024_64913_MOESM1_ESM.pdf]

Supplementary Materials

Semantic Segmentation

Semantic segmentation is a sub-field of computer vision and digital image processing that deals with the classification of images into pixel units, assigning each pixel to a specific object class. The cornerstone of semantic segmentation is the Fully- Convolutional Network (FCN) [1], which is a modified structure of convolutional neural network (CNN)-based models that have shown excellent performance in image classification. Most semantic segmentation models have been developed based on the FCN architecture. For the comparative experiments involving segmentation models, we selected for four models that are either widely employed within the medical domain or have demonstrated high performance. First, U-Net [2] is an end-to-end network designed for image segmentation in the biomedical field. It showed good performance with a small amount of data using an efficient augmentation technique in medical fields where data is difficult to secure. This network extracts image features using not only low- dimensional but also high-dimensional information, and uses a technique called skip connection to enable accurate localization. In addition, U-Net overcomes the trade-off by improving the slow computation speed and verifying the output of multiple layers simultaneously. Second, DeepLab V3+ [3] is an encoder-decoder network that utilizes Xception [4] as its backbone architecture. In the process of feature extraction by the encoder, DeepLab V3+ employs Atrous Spatial Pyramid Pooling (ASPP) [5] to capture multi-scale contextual information and achieve precise semantic segmentation. This technique enables a larger receptive field without increasing the number of parameters or computational complexity, thereby preserving the efficiency of conventional convolutions. Additionally, ASPP reduces the impact of model performance on object size by capturing information at multiple scales with only a single input image.

Next, HarDNet-MSEG [6] is a convolutional neural network designed for polyp segmentation. HarDNet-MSEG uses HarDNet68 [7] as the backbone network and is designed with encoder-decoder architecture to achieve high accuracy and efficient inference time. The cornerstone of HarDNet-MSEG is DenseNet [8], which increases inference speed by reducing shortcuts in DenseNet while increasing the width of the channel. In addition, to increase computational density, Harmonic Dense Block (HBD) forms a layer group followed by 1x1 convolution as a transition. Finally, like all previous networks, SegNet [9] is an encoder-decoder model that is widely used for road scene segmentation tasks. This network improved boundary delineation and reduced. the number of parameters efficiently by reusing the encoder's Max Pooling indices. Additionally, SegNet reduces computation costs such as memory usage by using a method that excludes the fully-connected layer.

Volume Estimation

From a clinical perspective, studies on volume measurements of various organs in the body, including the lungs, brain, and eyeball, are being conducted. Conventionally, volume measurements were performed based on formulaic calculations using various indicators or through the use of diverse program tools relying on medical imagery. Specifically in ophthalmology, estimation of eyeball volume was carried out using software like Terraricon, based on CT images, by simply measuring intraocular pressure. Additionally, volume estimation studies have been conducted not only for the eyeball but also for other ocular structures [10–12]. However, these volume measurement methods have inherent limitations in terms of accuracy and reproducibility, as results may vary depending on the medical clinicians performing the measurements. Depending on the measurement method, there were additional issues related to invasiveness and the risk of patient discomfort and infection.

To overcome the limitations of these existing methods, research on deep learning volume measurement methods for various organs in the body using medical image is steadily increasing [13–17]. In fact, due to the extremely small average volume of the eyeball, approximately 7cc, even minor segmentation errors can exert a significant influence on volume estimation performance. Additionally, there is the potential for various other factors to impact the accuracy of volume estimation. However, no matter how well the segmentation model performs, there can be significant variations in volume estimation performance. Therefore, for precise measurement of eyeball volume, careful consideration and refinement of both the segmentation model and the estimation method are imperative.

Table S1 The demographic statistics of CT dataset

Age(range)	Male (n, %)	Female (n, %)	Total (n, %)
29 and under	27 (13.5)	42 (21)	69 (34.5)
30 - 39	17 (8.5)	52 (26)	69 (34.5)
40 - 49	13 (6.5)	25 (12.5)	38 (19)
50 - 59	5 (2.5)	13 (6.5)	18 (9)
60 and over	3 (1.5)	3 (1.5)	6 (3)

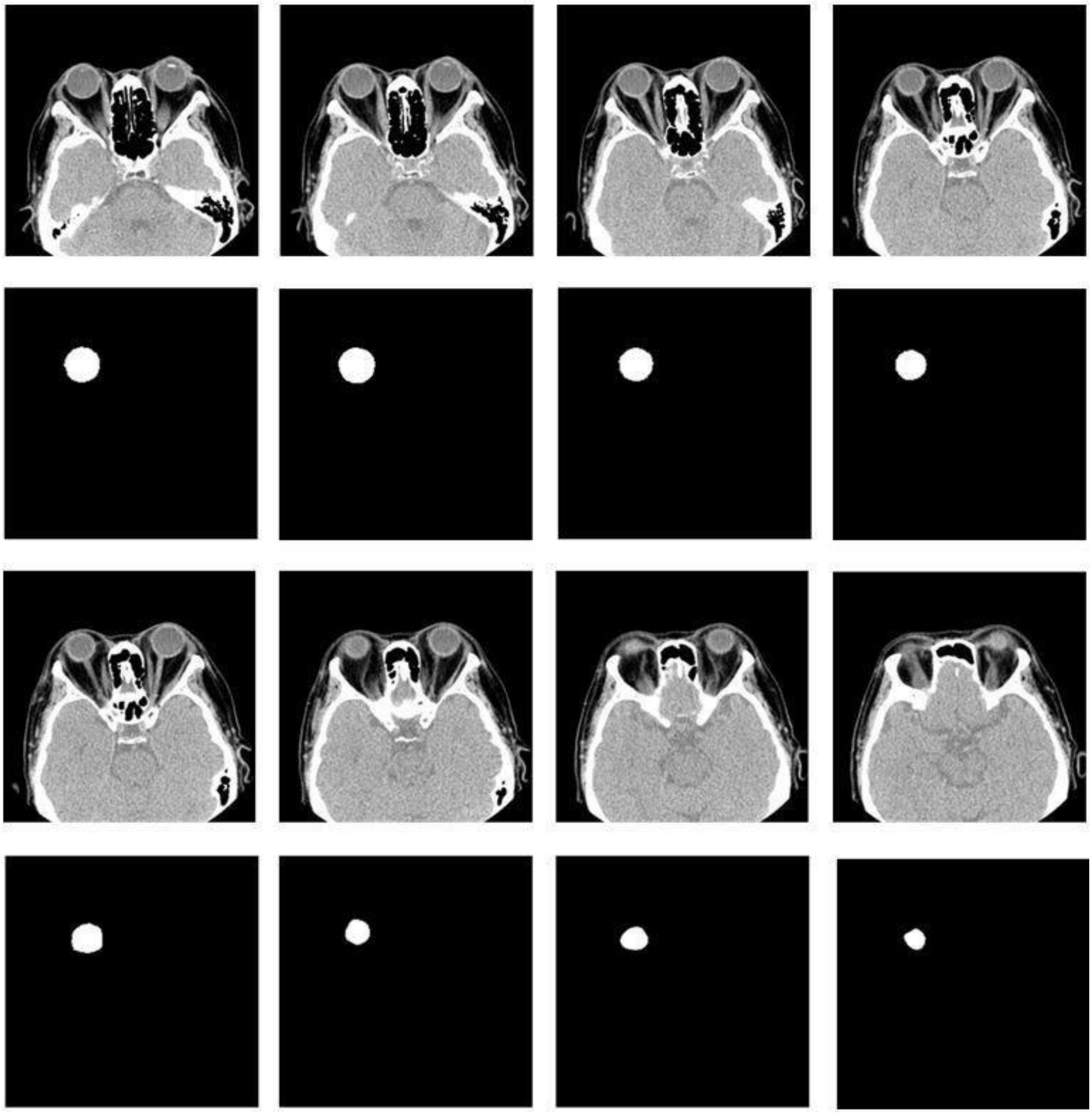

Figure S2 Example of segmentation dataset

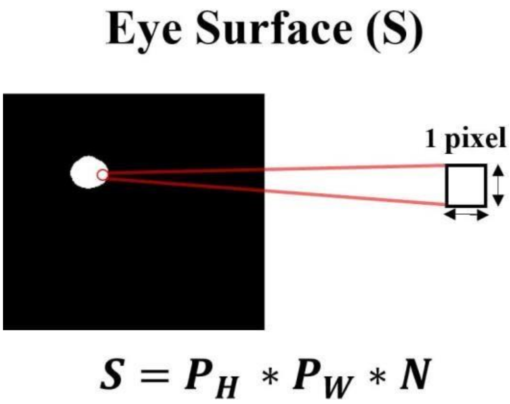

(a) Example of estimating the area from the mask image

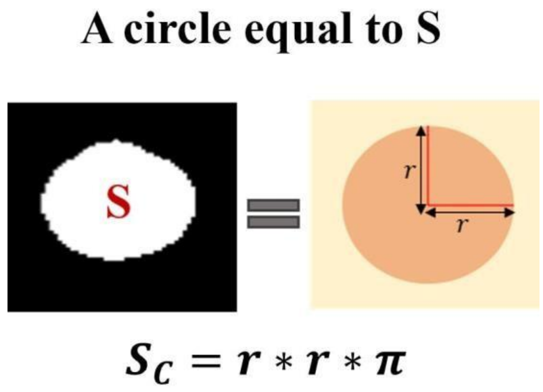

(b) Example of approximating the radius from the estimated area

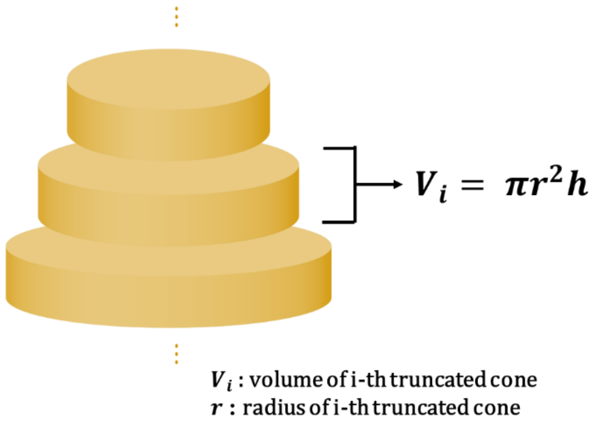

(c) Volume estimation method using the truncated cylinder formula

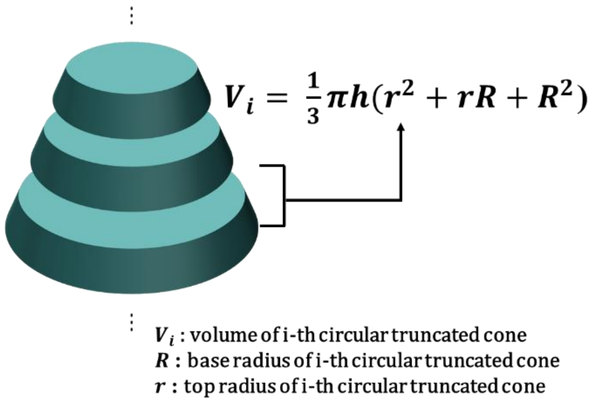

(d) Volume estimation method using the truncated cone formula

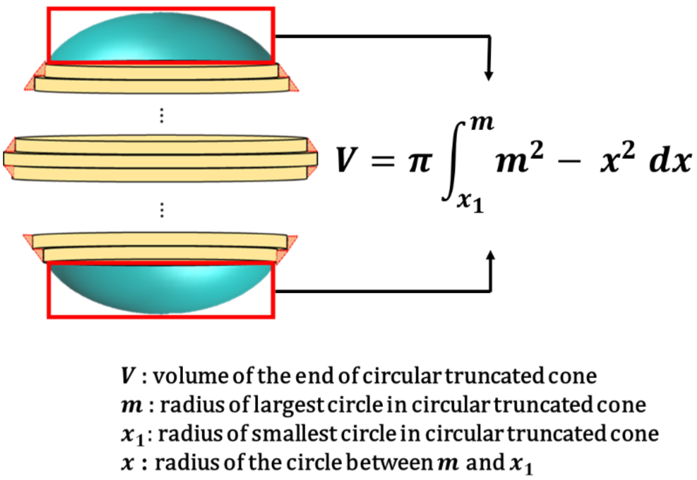

(e) The volume estimation method usingan integral equation reflecting prior information of eyeball shape

Figure S2. Subset process of volume estimation

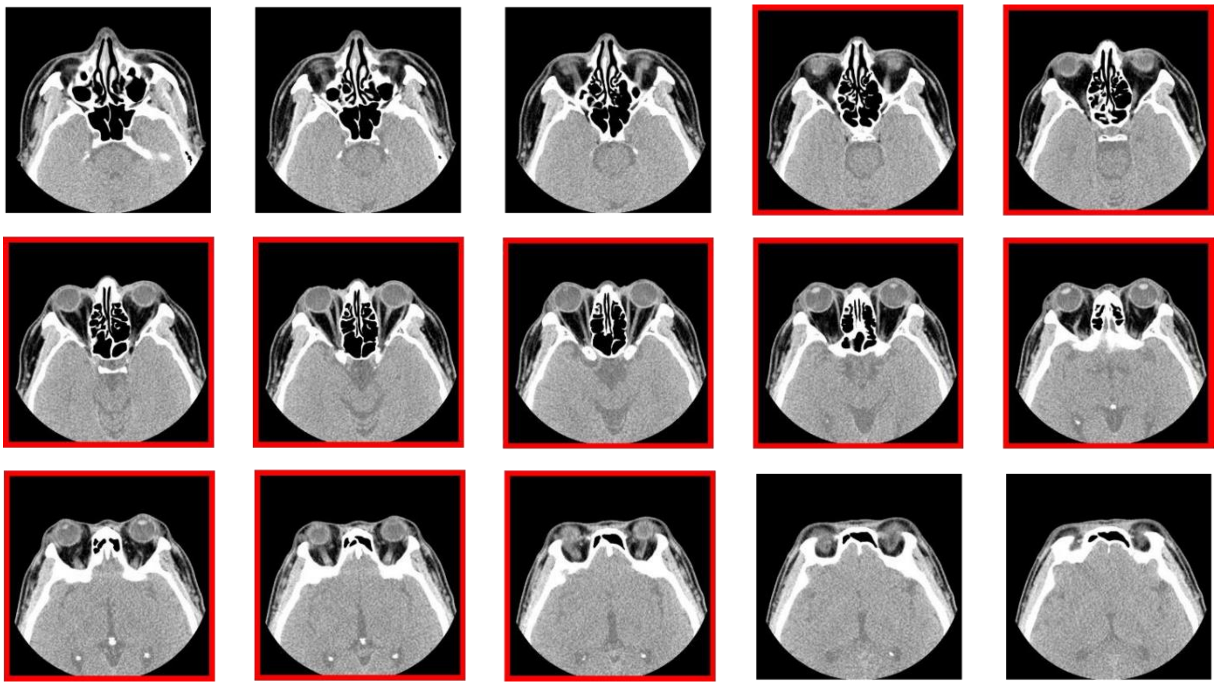

Figure S3. Example of CT slices from a random patient used for volume estimation. Red boxes indicate the specific slices used for volume estimation among all CT slices.

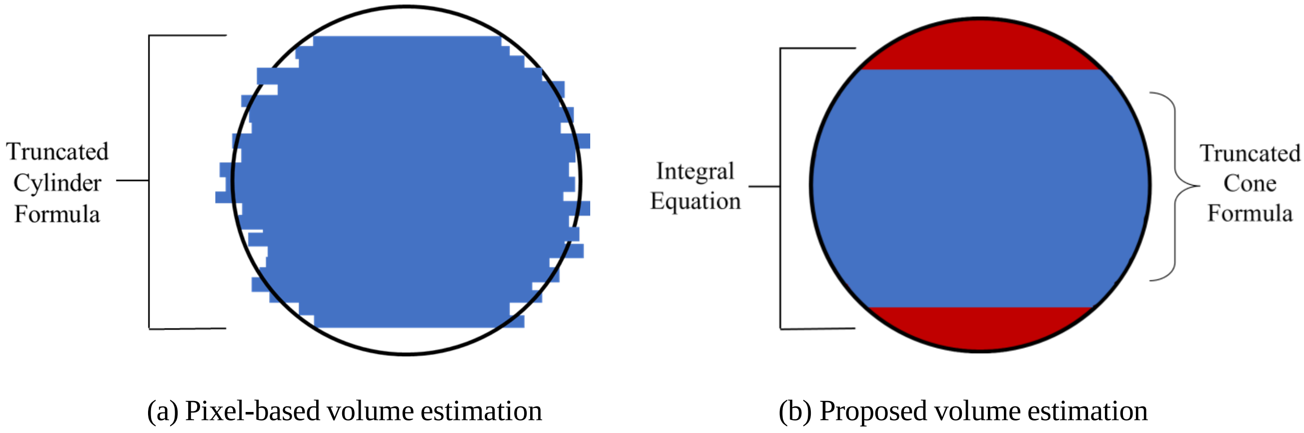

Figure S4. Comparison of volume estimation methods

References

[1] Long, J., Shelhamer, E., Darrell, T.: Fully convolutional networks for semantic segmentation. In: Proceedings of the IEEE Conference on Computer Vision and Pattern Recognition, pp. 3431–3440 (2015)

[2] Ronneberger, O., Fischer, P., Brox, T.: U-net: Convolutional networks for biomed- ical image segmentation. In: Medical Image Computing and Computer-Assisted Intervention–MICCAI 2015: 18th International Conference, Munich, Germany, October 5-9, 2015, Proceedings, Part III 18, pp. 234–241 (2015). Springer

[3] Chen, L.-C., Papandreou, G., Kokkinos, I., Murphy, K., Yuille, A.L.: Deeplab: Semantic image segmentation with deep convolutional nets, atrous convolution, and fully connected crfs. IEEE transactions on pattern analysis and machine intelligence 40(4), 834–848 (2017)

[4] Chollet, F.: Xception: Deep learning with depthwise separable convolutions. In: Proceedings of the IEEE Conference on Computer Vision and Pattern Recognition, pp. 1251–1258 (2017)

[5] Chen, L.-C., Zhu, Y., Papandreou, G., Schroff, F., Adam, H.: Encoder-decoder with atrous separable convolution for semantic image segmentation. In: Proceed- ings of the European Conference on Computer Vision (ECCV), pp. 801–818 (2018)

[6] Huang, C.-H., Wu, H.-Y., Lin, Y.-L.: Hardnet-mseg: A simple encoder-decoder polyp segmentation neural network that achieves over 0.9 mean dice and 86 fps. arXiv preprint arXiv:2101.07172 (2021)

[7] Chao, P., Kao, C.-Y., Ruan, Y.-S., Huang, C.-H., Lin, Y.-L.: Hardnet: A low mem- ory traffic network. In: Proceedings of the IEEE/CVF International Conference on Computer Vision, pp. 3552–3561 (2019)

[8] Huang, G., Liu, Z., Van Der Maaten, L., Weinberger, K.Q.: Densely connected convolutional networks. In: Proceedings of the IEEE Conference on Computer Vision and Pattern Recognition, pp. 4700–4708 (2017)

[9] Badrinarayanan, V., Kendall, A., Cipolla, R.: Segnet: A deep convolutional encoder- decoder architecture for image segmentation. IEEE transactions on pattern analysis and machine intelligence 39(12), 2481–2495 (2017)

[10] Lenchik, L., Heacock, L., Weaver, A.A., Boutin, R.D., Cook, T.S., Itri, J., Filippi, C.G., Gullapalli, R.P., Lee, J., Zagurovskaya, M., et al.: Automated segmentation of tissues using ct and mri: a systematic review. Academic radiology 26(12), 1695– 1706 (2019)

[11] Furuta, M.: Measurement of orbital volume by computed tomography: especially on the growth of the orbit. Japanese journal of ophthalmology 45(6), 600–606 (2001)

[12] Tandon, R., Aljadeff, L., Ji, S., Finn, R.A.: Anatomic variability of the human orbit. Journal of Oral and Maxillofacial Surgery 78(5), 782–796 (2020)

[13] Jafrasteh, B., Lubi ́an-L ́op ez, S.P., Benavente-Fern ́andez, I.: A deep sift convo- lutional neural networks for total brain volume estimation from 3d ultrasound images. Computer Methods and Programs in Biomedicine, 107805 (2023)

[14] Jaichandran, R., et al. "Brain Tumour Segmentation and Volume Estimation using Efficient Convolution Neural Network for MRI Images." 2023 International Conference on Distributed Computing and Electrical Circuits and Electronics (ICDCECE). IEEE, 2023.

[15] Hobday, H., Cole, J.H., Stanyard, R.A., Daws, R.E., Giampietro, V., O'Daly, O., Leech, R., V ́aˇs a , F.: Tissue volume estimation and age prediction using rapid structural brain scans. Scientific Reports 12(1), 12005 (2022)

[16] Gerard, S.E., Chaudhary, M.F., Herrmann, J., Christensen, G.E., San Jose Estepar, R., Reinhardt, J.M., Hoffman, E.A.: Direct estimation of regional lung volume change from paired and single ct images using residual regression neural network. Medical physics (2023)

[17] Liao, F., Chen, X., Hu, X., Song, S.: Estimation of the volume of the left ventricle from mri images using deep neural networks. IEEE transactions on cybernetics 49(2), 495–504 (2017)
